# Copy number variation in Han Chinese individuals with autism spectrum disorder

**DOI:** 10.1186/1866-1955-6-34

**Published:** 2014-08-23

**Authors:** Matthew J Gazzellone, Xue Zhou, Anath C Lionel, Mohammed Uddin, Bhooma Thiruvahindrapuram, Shuang Liang, Caihong Sun, Jia Wang, Mingyang Zou, Kristiina Tammimies, Susan Walker, Thanuja Selvanayagam, John Wei, Zhuozhi Wang, Lijie Wu, Stephen W Scherer

**Affiliations:** 1 The Centre for Applied Genomics and Program in Genetics and Genome Biology, The Hospital for Sick Children, 686 Bay Street, Peter Gilgan Centre for Research and Learning, Room 139800, Toronto, Ontario M5G 0A4, Canada; 2 Department of Molecular Genetics and McLaughlin Centre, University of Toronto, Toronto Ontario M5S 1A1, Canada; 3 Department of Children’s and Adolescent Health, Public Health College of Harbin Medical University, Harbin, Heilongjiang 150086, People’s Republic of China; 4 Heilongjiang Provincial Centre for Disease Control and Prevention, Harbin, Heilongjiang 150030, People’s Republic of China; 5 Center of Neurodevelopmental Disorders, Department of Women’s and Children’s Health, Karolinska Institutet, Stockholm 113 30, Sweden

**Keywords:** Autism spectrum disorder (ASD), Copy number variations (CNVs), Microarray diagnostic testing, Han Chinese

## Abstract

**Background:**

Autism spectrum disorders (ASDs) are a group of neurodevelopmental conditions with a demonstrated genetic etiology. Rare (<1% frequency) copy number variations (CNVs) account for a proportion of the genetic events involved, but the contribution of these events in non-European ASD populations has not been well studied. Here, we report on rare CNVs detected in a cohort of individuals with ASD of Han Chinese background.

**Methods:**

DNA samples were obtained from 104 ASD probands and their parents who were recruited from Harbin, China. Samples were genotyped on the Affymetrix CytoScan HD platform. Rare CNVs were identified by comparing data with 873 technology-matched controls from Ontario and 1,235 additional population controls of Han Chinese ethnicity.

**Results:**

Of the probands, 8.6% had at least 1 *de novo* CNV (overlapping the *GIGYF2*, *SPRY1*, 16p13.3, 16p11.2, 17p13.3-17p13.2, *DMD*, and *NAP1L6* genes/loci). Rare inherited CNVs affected other plausible neurodevelopmental candidate genes including *GRID2*, *LINGO2*, and *SLC39A12*. A 24-kb duplication was also identified at *YWHAE*, a gene previously implicated in ASD and other developmental disorders. This duplication is observed at a similar frequency in cases and in population controls and is likely a benign Asian-specific copy number polymorphism.

**Conclusions:**

Our findings help define genomic features relevant to ASD in the Han Chinese and emphasize the importance of using ancestry-matched controls in medical genetic interpretations.

## Background

Autism spectrum disorders (ASDs) are characterized by impairments in communication, social interaction, as well as repetitive and restrictive behaviors. Individuals with ASDs also frequently demonstrate other medical challenges such as intellectual disability and seizures, and the ASD phenotype is recognized in over 100 different medical genetic disorders [1]. Underlying genetic risk factors, particularly rare penetrant genic copy number variations (CNVs), are thought to explain approximately 5–10% of the disorder depending on the cohort examined. However, no single candidate genetic locus has been implicated in more than 1% of ASD cases and hundreds of candidate genes have been identified [1].

Most CNV studies in ASD have focused on individuals of European ancestry [2-4], but studies describing the genetic architecture in other populations are also required, particularly as clinical microarray testing continues to be adopted as the standard of care across medical genetic labs worldwide [5]. Outside North America and Western Europe, the reported prevalence of ASD varies from the approximately 1% usually noted [6]. In China, the first ASD cases were not detected until the early 1980s and a meta-analysis estimated the rate of ASD in newborns as 11.8 per 10,000 [7].

Genetic studies of ASD in Han Chinese individuals have been presented primarily as case reports or association studies of particular common single nucleotide polymorphisms (SNPs) previously identified in individuals with ASD of European ancestry [8]. So far, however, there has been a dearth of published studies of rare CNVs in Chinese autism cohorts. Moreover, previous CNV studies in the general Asian population have shown substantial differences in terms of location and frequency of some CNVs [9], necessitating our ongoing work to examine the characteristics of CNVs in a Han Chinese cohort with clinically diagnosed ASD.

## Methods

### Sample selection

ASD-affected individuals and their families were referred to the Children Development and Behavior Research Center (CDBRC) at Harbin Medical University, China, by their community physician between January 2007 and June 2011. Proband diagnosis and study inclusion criteria were completed as previously described [8]. The Autism Behavior Checklist (ABC) and Childhood Autism Rating Scale (CARS) were used for diagnosis. We report on 104 consecutive cases with an ASD diagnosis made by two psychiatrists at the CDBRC. Subsequently, DNA was also obtained from the parents of these ASD individuals. Proband participants consisted of 91 males (87.5%) and 13 females (12.5%). The mean age of the probands at enrolment was 4.31 ± 1.80 years. The study was approved by the Ethics Committee at Harbin Medical University and written consent was obtained from parents.

### Genotyping and variant calling

Genotyping was performed on the Affymetrix CytoScan HD platform (Santa Clara, CA, USA) according to the manufacturer's specifications. PLINK software was used to confirm the Han Chinese ethnicity of all individuals in the study from extracted SNP genotypes (Additional file 1: Figure S1). Samples from 100 probands and 200 parents passed our quality control metrics including 93 complete trios. CNVs were called via our CNV detection pipeline (Additional file 1: Figure S2). Population control datasets used to distinguish rare variants included three Han Chinese-specific cohorts and one microarray-specific set of primarily European individuals (see Additional file 1). For all case and control samples, genotyping and CNV calling were performed using identical procedures [10] and CNV data was compared against the Database of Genomic Variants [11]. Validation methods confirmed 96% (27/28) of the CNVs tested.

### Expression analysis

The SuperScript III First-Strand Synthesis SuperMix for qRT-PCR kit (Life Technologies, Carlsbad, CA, USA) was used to generate cDNA from RNA extracted from 11 tissues and a whole brain sample. Expression analysis and tissue distribution of *CASKIN1* and *PKD1* were illustrated using quantitative RT-PCR. The housekeeping genes *MED13* and *ACTB* were used to normalize the expression level.

## Results

Using a high-resolution CNV genotyping array and a well-established CNV calling protocol (Additional file 1: Figure S2), we identified 241 rare CNVs in our probands (Additional file 1: Table S1). We established that 11 of the probands from the 93 complete trios (11.8%) carried a *de novo* or rare inherited CNV that may contribute to ASD (Table 1). The CNV profiles of both the ASD cases and their parents indicated that there is no significant difference in the overall CNV call rates or the length of CNVs between these two groups (Additional file 1: Table S2).

**Table 1:** **Summary of *de novo *** **and rare inherited CNVs of interest in ASD probands**

**Sample ID**	**Cytoband**	**Coordinates (hg19)**	**Type of CNV**	**Genes affected**	**Inheritance**
683-3 (female)	2q37.1	Chr2: 233,651,280-233,673,273	22-kb deletion	*GIGYF2*	*De novo*
527-3 (male)	4q28.1	Chr4: 124,063,146-125,045,116	982-kb duplication	*SPRY1*, *SPATA5*	*De novo*
517-3 (female)	16p13.3	Chr16: 843,861-1,162,728	319-kb duplication	7 genes	*De novo*
	16p13.3	Chr16: 2,088,391-2,415,016	327-kb duplication	15 genes	*De novo*
503-3 (male)	16p11.2	Chr16: 28,819,029-29,051,191	232-kb deletion	9 genes	*De novo*
692-3 (male)	17p13.3, 17p13.2	Chr17: 2,455,643-3,449,869	994-kb duplication	16 genes	*De novo*
567-3 (male)	Xp21.1	ChrX: 31,805,650-31,959,887	154-kb deletion	*DMD*	*De novo*
611-3 (male)	Xp21.1	ChrX: 32,548,066-32,603,018	55-kb deletion	*DMD*	*De novo*
552-3 (male)	Xq13.2	ChrX: 72,319,907-72,353,391	33-kb deletion	*NAP1L6*	*De novo*
694-3 (male)	4q22.2	Chr4: 94,144,621-94,172,410	28-kb deletion	*GRID2*	Maternal
	9p21.1	Chr9: 28,491,679-28,630,598	139-kb deletion	*LINGO2* (intronic)	Paternal
511-3 (male)	9p21.1	Chr9: 28,464,218-28,596,286	132-kb deletion	*LINGO2*	Maternal
686-3 (male)	10p12.33	Chr10: 18,240,592-18,313,842	73-kb deletion	*SLC39A12*	Paternal

All CNVs shown above have been confirmed via qPCR or a targeted TaqMan assay, and *de novo* status and inheritance was verified by testing both parental samples using these same methods.

### *De novo* variants

Nine *de novo* CNV events were detected in eight probands (Table 1). This represents a *de novo* CNV rate of 8.6% (8/93), similar to that which has been seen previously in other non-Chinese CNV studies [2-4,12]. In one female proband (683-3), we discovered a 22-kb deletion at 2q37.1 overlapping *GIGYF2*, which lies in a susceptibility locus for familial Parkinson's disease [13]. No ASD phenotype has been previously associated with variants affecting this gene.

A 982-kb *de novo* duplication was uncovered in male proband 527-3. This duplication overlaps *SPRY1*, a gene that regulates fibroblast growth factor signaling which plays an important role in the patterning and propagation of cells in the developing brain [14]. There is no prior evidence of any link between duplications overlapping this gene or *SPATA5* and ASD.

A pair of adjacent *de novo* duplications at 16p13.3 separated by nearly 1 Mb of two-copy intervening sequence was uncovered in a 6-year-old female (517-3). To better assess which genes might be contributing to the phenotype, we referred to a recent finding showing that highly brain-expressed exons have a lower burden of rare missense variants than more ubiquitously expressed exons and are targets for penetrant mutations in ASD [15]. We then checked each of the exons in the genes residing within the duplications to determine if any of these were characterized as a ‘brain-critical exon.’ This was the case for at least one exon in *RAB26*, *PKD1*, *E4F1*, *ABCA3*, and *CASKIN1* (Additional file 1: Figure S3). We focused on *CASKIN1* and *PKD1* due to previous studies indicating that they may play some role in synaptic scaffolding and neurodevelopment [16,17]. In these two genes, we confirmed the presence of a brain-expressed isoform whose dosage could be impacted by the duplication (Additional file 1: Figure S3). The 16p13.3 duplication affects a different region of that chromosomal band than was recently described to be involved in obsessive-compulsive disorder [18].

A male proband (503-3) was found to carry a 232-kb *de novo* microdeletion at 16p11.2. This deletion lies upstream of the 600-kb ASD-implicated risk locus [1]. Similarly sized deletions have been previously noted in individuals with obesity and developmental delay [12,19]. Unlike many of the individuals with similar deletions, this male has a BMI of 18.26, putting him in the normal range.

A 994-kb duplication of 17p13.3-17p13.2 was uncovered in a 5-year-old autistic male (692-3). Microduplications have been previously noted at this locus and are usually associated with developmental delay and frequently with growth issues [20]. This proband has typical autistic features and no evidence of any growth problems.

Two unrelated male probands harbor *de novo* exonic deletions of the Duchenne Muscular Dystrophy (*DMD*) gene. The first case (611-3), a 5-year-old autistic male presenting with ASD, hypotonia, and progressive motor impairments including difficulty walking, has a 55-kb deletion that overlaps exons 14–17. The second proband (567-3), an autistic 6-year-old male with abnormal muscular development, has a deletion of 154 kb which overlaps exons 46–50. In both cases, the deletion is predicted to cause a frameshift leading to a premature stop and loss of dystrophin. In males, such mutations are predicted to result in DMD. Studies have shown a higher incidence of ASD in boys with DMD, possibly because of a secondary synaptic role for the protein [21].

A 33.5-kb deletion at Xq13.2 has also been noted in male case 552-3. This deletion overlaps the *NAP1L6* nucleosome assembly protein. There are no published reports of deletions affecting this gene.

### Rare inherited variants

We identified a 27.8-kb loss of two exons of *GRID2* in a 4-year-old male (694-3). *GRID2* encodes a glutamate receptor channel subunit and mutations within *GRID2* have been associated with ASD [2]. Our proband inherits the mutation from his mother who has a diagnosis of intellectual disability. His maternal grandfather and a maternal cousin also have intellectual disability, but no DNA was available to test the segregation of this variant in these individuals.

A 73-kb deletion affecting *SLC39A12* was found in a 6-year-old male proband (686-3). All but the last exon of the gene was deleted in the proband and his unaffected father. A recent study suggests that this zinc transporter stimulates neurite outgrowth during neurodevelopment [22].

Finally, we identified a male proband (511-3) with a maternally inherited deletion of one exon of *LINGO2*. The gene has been previously associated with adult-onset neurodegenerative disorders and has been implicated as an ASD risk gene [23]. A second male proband (694-3) in our study also harbors an intronic CNV within this gene, but its potential effect on gene expression and any possible contribution to phenotype were not possible to ascertain.

### Population-specific CNV polymorphisms

**Figure 1:**
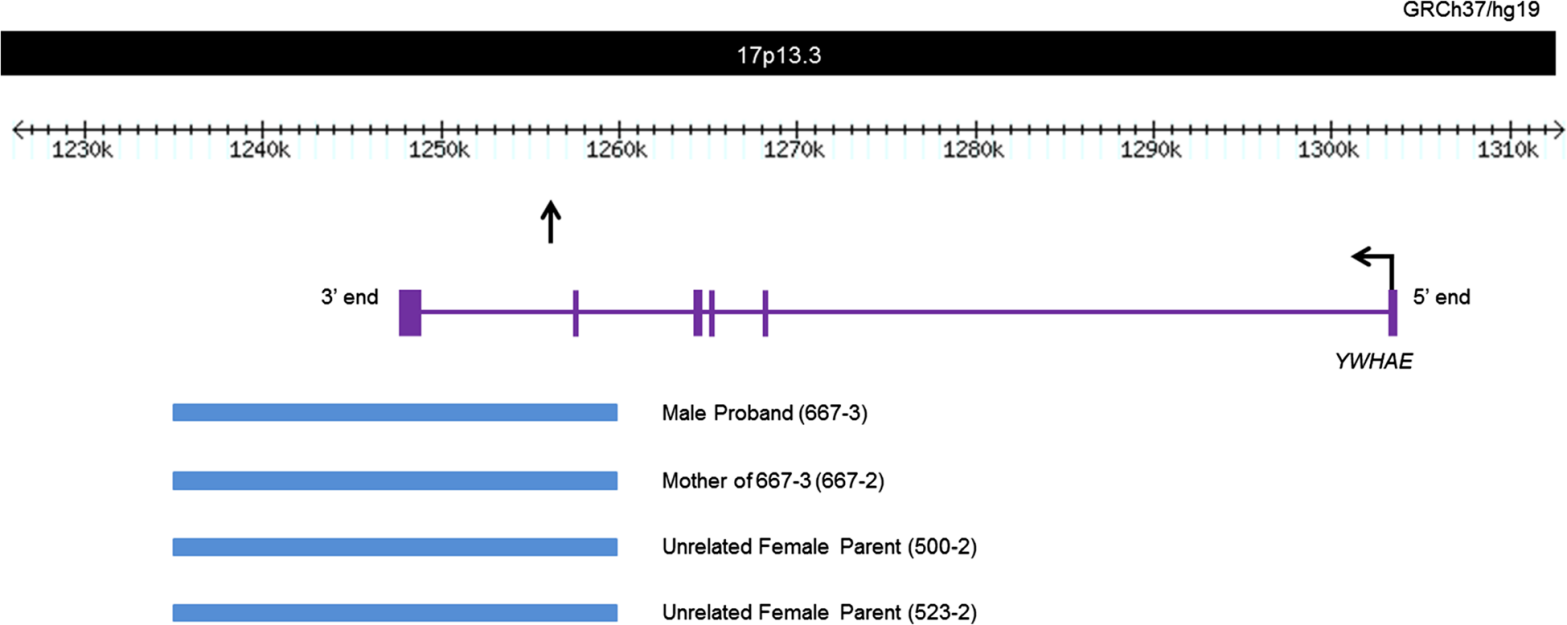
**Genomic location of *YWHAE *duplications.** The locations of the duplications (represented by *blue bars*) in the four individuals tested overlapping the 3′ end of *YWHAE* locus are shown. We have also identified 11 similarly sized duplications in Chinese control samples (from samples run on different arrays). Three additional duplications in a second cohort of Chinese population controls were also found at this locus by screening using a quantitative assay (the site of the TaqMan Copy Number Assay is indicated by the *vertical arrow*).

We have also identified a population-specific CNV polymorphism in *YWHAE*, a gene previously speculated to have a role in ASD [20]. Here, we identified a 24-kb duplication (chr17: 1,235,975-1,259,833) overlapping the last exon of this gene in one proband and his mother. No microduplications of *YWHAE* were found in Caucasians in our Ontario population controls. However, we identified similar duplications in two unrelated parents of other probands in this Han Chinese cohort (Figure 1). We attempted fine-mapping of the breakpoints in the four different samples carrying the rearrangement and found that both the 3′ and 5′ ends consistently map to the same regions, suggesting that the CNVs likely represent the same ancestral event. We were unable to determine the precise sites of the breakpoints due to complex sequence elements located in the region. Subsequently, we assessed the frequency of this event in the Chinese population and found that 11/1,235 (0.9%) of the Han Chinese population controls carried a microduplication at this locus. Using a TaqMan Copy Number Assay for a probe located within the breakpoints of the microduplication, we found duplications in 3/260 additional Han Chinese controls. In all, approximately 1% of Han Chinese individuals have this duplication, regardless of ASD status. We notice no statistically significant difference between the frequency of this variant in cases versus controls (*p* = 1.000 using a two-tailed Fisher's exact test).

## Discussion

Our data adds to the growing number of CNV studies in autism, all of which are critical for accurate interpretation of post- and pre-natal testing being performed or contemplated in the clinical genetic setting [5,24]. We have confirmed the important contribution of *de novo* and rare inherited CNVs in ASD. Though many of the genes and loci that have been identified in this study have been previously detected in European individuals, population-specific variants overlapping ASD candidate genes were identified in cases and Han Chinese population controls. This reaffirms the need to account for ancestry for the most accurate interpretation of clinical microarray data. We anticipate that as CNV studies become routine in other ASD populations, we will discover additional examples of population-specific CNVs, which are overlapping ASD candidate genes, but are non-pathogenic [25].

There was no difference with respect to CNV call rate or the length of CNVs between probands and their parents in our study. However, some groups that have examined larger cohorts using microarrays [2] or analyzed smaller types of variation using exome sequencing [26] have noted a greater enrichment of CNVs in cases when compared to controls. Additional high-resolution studies of copy number variation in sufficiently large ASD sample cohorts will help further clarify potential CNV enrichment in ASD.

Perhaps our most important finding in this paper is the identification of the *YWHAE* CNV that appears to be a Chinese-specific polymorphism and not an ASD (or developmental delay)-associated variant. The gene product, 14-3-3ϵ, is a member of the 14-3-3 family of genes which are highly conserved across species and play an important role in protein regulation and signal transduction [27]. Changes in 14-3-3ϵ dosage have been shown to alter neuronal migration, thereby impairing proper neurodevelopment [27]. Several studies have previously indicated that large duplications or deletions at 17p13.3 affecting this gene are associated with developmental delay or autism [20,28-30]. The small 24-kb duplication that we identified in our study has also been noted in our past work [31] and in clinical cohorts. In light of these findings, we reevaluated all cases with the 24-kb duplication and found that they clustered with Asian individuals when determining ancestry from extracted SNPs. Unlike the larger CNVs previously identified at this locus [20,28-30], this small duplication is not likely to be pathogenic since the frequency of the event is similar in cases and in population controls. Our finding with *YWHAE* provides an example of the importance of using ancestry-matched controls when characterizing the clinical relevance of rare variants in a population.

The prevalence of ASD in mainland China and Western countries varies considerably [1,6,7]. Our exploratory study of the role of CNVs in ASD families in China does not yet provide a genetic explanation for such differences, and it is more likely that ascertainment bias is involved. Specifically, it is hypothesized that differences in assessment protocols and cultural expectations (especially with regard to eye contact and speech) could influence the recognition of autistic behaviors in ASD individuals with normal or mildly impaired cognitive functioning [6,7]. Moreover, parental attitudes and a stigma surrounding neuropsychiatric conditions may also contribute to a reticence towards accepting a potential autism diagnosis [7], possibly precluding an affected child from being enrolled in any study of ASD.

The results of this study substantiate the extensive genetic heterogeneity that is inherent to autism. As a result, it is of great importance to examine the totality of genetic variation in order to accurately identify new, potentially causal genes. Though whole-exome and whole-genome technologies already identify interesting single nucleotide variants and show promise in detecting in/dels and copy number changes, microarrays still remain the gold standard for CNV detection. Future CNV studies of Han Chinese individuals can build upon the foundation established by this study. Further work in this population can identify new population-specific variants that may contribute to ASD, as well as add further support for the contribution of rare CNVs to ASD.

## Conclusions

Our study serves as a pilot to provide initial insight into the genetic architecture of ASD in the Han Chinese population. Ongoing genome sequencing experiments indicate that many more etiologic genetic variants will be found with higher-resolution technologies [32]. At this time, CNV testing in the Han Chinese population using microarrays would be most appropriate usually in a confirmatory diagnostic setting, at least until larger cohorts of matched control data become available for comparisons.

## Abbreviations

ABC: Autism Behavior Checklist; ASD: autism spectrum disorder; CARS: Childhood Autism Rating Scale; CDBRC: Children Development and Behavior Research Center; CNV: copy number variation.

## Competing interests

The authors declare that they have no competing interests.

## Authors’ contributions

MJG and XZ analyzed the microarray calls and performed experimental validation. MJG, ACL, MU, SW, TS, and KT contributed to the interpretation of the results and completed additional experimental analyses. BT, JW, and ZW were involved with the informatics analyses. WJ, SC, and LS collected and evaluated the cases. ZM performed the DNA extraction. MJG and SWS wrote the manuscript. LW and SWS supervised the project. All authors read and approved the final manuscript.

## Authors’ information

Lijie Wu and Stephen W Scherer are co-senior corresponding authors.

## Supplementary Material

Additional file 1 **Supplemental methods. Figure S1.** Ancestry determination in our ASD cohort. **Figure S2.** CNV detection workflow. **Figure S3.** Identification of brain-critical exons at 16p13.3. **Table S1.** List of rare CNVs. **Table S2.** Summary statistics of stringent CNVs larger than 20 kb. Click here for file
