# Supplementary material for: Copy number variation in Han Chinese individuals with autism spectrum disorder
Source: J Neurodev Disord. 2014 Aug 23;6(1):34. doi: 10.1186/1866-1955-6-34 (PMC4147384; doi:10.1186/1866-1955-6-34)
Supplement: Additional file 1 — Supplemental methods. Figure S1. Ancestry determination in our ASD cohort. Figure S2. CNV detection workflow. Figure S3. Identification of brain-critical exons at 16p13.3. Table S1. List of rare CNVs. Table S2. Summary statistics of stringent CNVs larger than 20 kb. [file 1866-1955-6-34-S1.pdf]

Supplemental Methods

Microarray Genotyping and Quality Control

DNA samples were submitted to The Centre for Applied Genomics in Toronto and genotyping was performed on the Affymetrix CytoScan HD platform. For arrays to be considered in the analysis, runs were required to meet or exceed the quality control thresholds specified by Affymetrix. These included a MAPD (Median Absolute Pairwise Difference) of ≤0.25, a SNP QC (SNP Quality Control) of ≥15.0, and a Waviness SD (Waviness Standard Deviation) of ≤0.12. In total, 104 probands and 206 parents met or exceeded these minimum criteria. DNA samples from two of the mothers failed to meet the appropriate thresholds and were excluded from the rest of the study.

Detection and Characterization of CNVs

Four different CNV detection algorithms were used to limit false-positive detections and generate high-confidence calls. These algorithms included the Affymetrix Chromosome Analysis Suite (ChAS) (Affymetrix Inc., USA), iPattern [1], Nexus [2], and Partek [3]. CNVs were required to span a minimum of 5 consecutive microarray probes and 20 kb. Only those CNVs that were defined as "stringent" were subsequently analyzed. A stringent CNV implies that it was detected by one or both of ChAS or iPattern, and if detected by only one of these algorithms, also by one of Nexus or Partek. Stringent calls on the X chromosome required calling by both ChAS and iPattern. Any sample whose total number of calls exceeded the mean by three or more times the standard deviation was removed from further analysis. A total of 100 probands and 200 parents (totaling 93 complete trios) passed these quality metrics. We confirmed the parentage for the 93 probands that were part of a trio using the PLINK tool set [4]. Rare CNVs could not overlap CNVs found at a frequency of greater than 0.1% in the control cohorts and were required to be at least 50% unique by length.

Population-based control datasets

Four population-based datasets were used as controls in this study. The first included 873 DNA samples obtained as part of the Ontario Population Genomics Platform (OPGP) [5]. These samples were genotyped on the Affymetrix CytoScan HD array in the same fashion as the ASD cases and analyzed for CNVs using the same methods as those obtained from the ASD cohort. Over 95% of the samples in this cohort were obtained from individuals of European ancestry. These samples were used as our primary control dataset for use in the detection of rare copy number changes and to account for calling biases inherent to this array. The other three datasets consisted of individuals of Han Chinese ethnicity and comprised a secondary set by which we could detect rare CNVs specific to the Han Chinese population. The first included 170 Han Chinese individuals from the HapMap project [6]. The second included 147 Han Chinese controls genotyped by Lou et al [7]. Both of these datasets were run on the Affymetrix 6.0 array. The third dataset contained 918 Han Chinese samples collected as part of the Singapore Genome

Variation Project [8]. These samples were genotyped on the Illumina 1M Duo array. The use of these different cohorts enables the distinction of those CNVs that are truly rare in the Chinese population as opposed to rare only when compared to samples obtained from European individuals.

Experimental Validation of CNV calls

CNVs of interest were validated using SYBR-Green based real-time quantitative PCR (qPCR) where possible. Primers were designed to amplify a region 90-140 bp in size using Primer3 software v. 0.4.0 (http://bioinfo.ut.ee/primer3-0.4.0/). Control primers were designed within the *FOXP2* locus which was used as a diploid locus control (Primer sequences available by request). For CNVs of interest, proband and paternal DNA were interrogated using this method. In addition, two HapMap samples (NA15510 (Female) and NA10851 (Male)), were used as "normal copy number" controls. All experiments were performed in triplicate. A TaqMan Copy Number Reference Assay was used to confirm additional copy number changes found in probands and their parents. Predesigned probes were selected within the target regions of interest and a reference assay amplified a two copy region. Both NA15510 (Female) and NA10851 (Male) were used as two-copy controls for autosomal CNVs.

Supplementary Figure 1: Ancestry Determination in our ASD Cohort

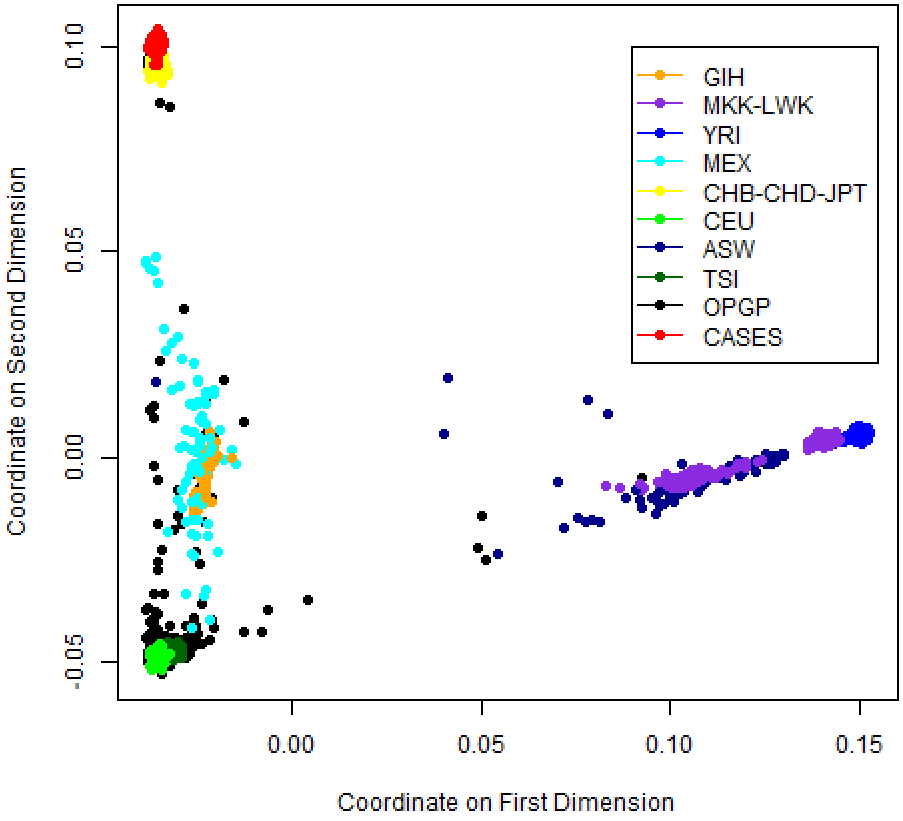

This plot was generated using 122,368 autosomal SNPs common to both the Affymetrix 6.0 and CytoScan HD platforms. All SNPs were required to have a genotyping rate exceeding 95% and a minor allele frequency greater than 5%. The plot shows that our sample cohort clusters with individuals of Asian ethnicity while our OPGP controls cluster primarily with individuals of European ancestry. This illustrates the importance of using additional sets of Han Chinese controls when identifying rare CNVs.

Supplementary Figure 2: CNV Detection Workflow

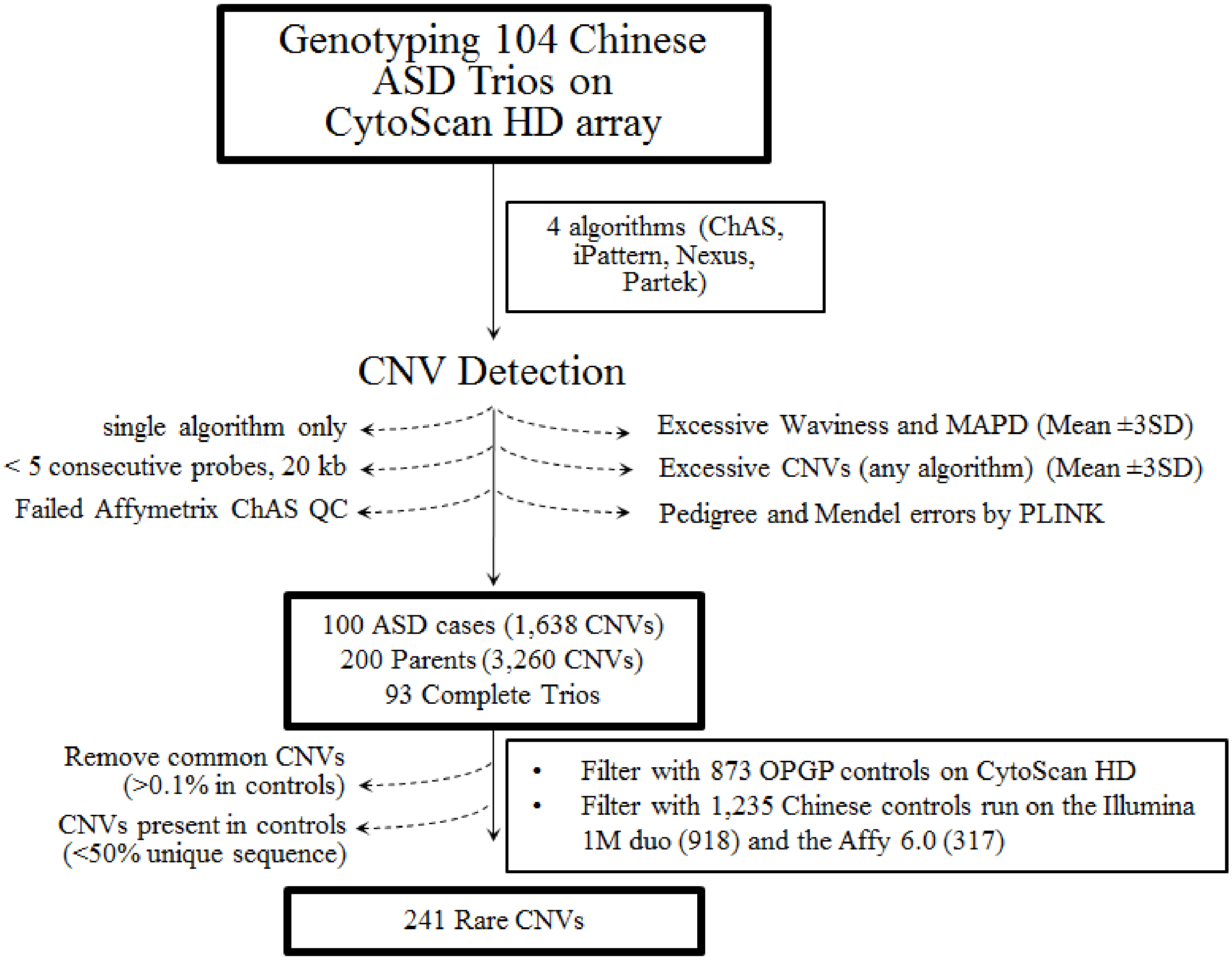

Supplementary Figure 3: Identification of Brain-Critical Exons at 16p13.3

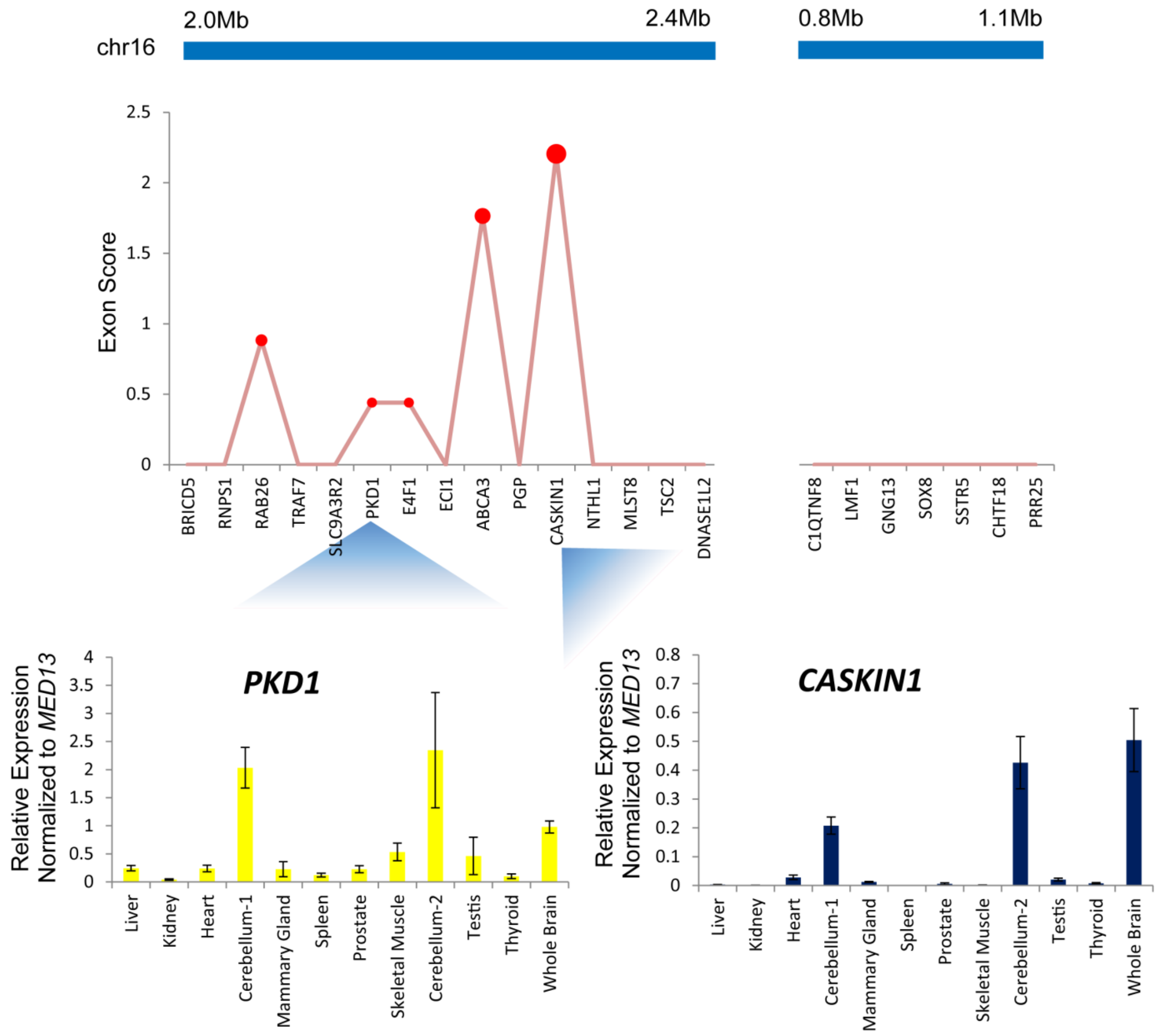

We computed the exon score as described previously [9] for each exon within the 16p13.3 duplications in our female proband to identify "brain-critical exons". Of the five genes scoring above zero, *CASKIN1* and *PKD1* were selected for further investigation. We show that expression of these exons within these genes is higher in cerebellum and whole brain using a quantitative real-time PCR assay which looked at relative expression in these genes compared to *MED13* (*ACTB* was used instead in a confirmatory assay).

Supplementary Table 1: List of Rare CNVs

Sample ID	chr	start	end	size	Sex	CNV	genes
666-3	10	7,196,973	7,315,361	118,388	M	Deletion	SFMBT2
666-3	12	96,014,096	96,706,369	692,273	M	Duplication	CCDC38,HAL,ELK3,NTN4,CDK17,AMDHD1,LTA4H,SNRPF
666-3	3	55,284,137	55,403,172	119,035	M	Deletion	-
666-3	4	56,756,201	56,780,082	23,881	M	Duplication	EXOC1
666-3	Y	25,415,633	25,618,373	202,740	M	Deletion	DAZ3,DAZ2,DAZ4
556-3	19	17,492,673	17,683,241	190,568	M	Duplication	TMEM221,PGLS,COLGALT1,FAM129C,BST2,NXNL1, MVB12A,SLC27A1
556-3	2	35,142,821	35,431,860	289,039	M	Deletion	-
556-3	20	23,836,569	23,924,631	88,062	M	Duplication	CST5
556-3	4	38,798,854	38,829,628	30,774	M	Deletion	TLR1,TLR6
684-3	2	26,621,755	26,648,953	27,198	M	Deletion	DRC1
632-3	3	106,837,905	106,869,829	31,924	F	Deletion	-
632-3	7	1,169,619	1,192,647	23,028	F	Deletion	C7orf50,ZFAND2A
527-3	4	124,063,146	125,045,116	981,970	M	Duplication	SPRY1,SPATA5
527-3	8	8,541,144	8,566,235	25,091	M	Deletion	CLDN23
527-3	Y	3,951,652	4,159,244	207,592	M	Deletion	-
690-3	17	28,937,651	29,140,813	203,162	M	Duplication	CRLF3
690-3	3	19,672,864	19,795,693	122,829	M	Deletion	-
690-3	Y	19,797,643	19,962,926	165,283	M	Duplication	XKRY,XKRY2
690-3	Y	20,212,995	20,381,761	168,766	M	Duplication	XKRY,XKRY2
505-3	13	43,587,160	43,608,103	20,943	M	Duplication	DNAJC15
505-3	6	162,290,899	162,359,917	69,018	M	Duplication	PARK2
505-3	7	31,681,765	31,921,749	239,984	M	Duplication	PDE1C,PPP1R17,CCDC129
505-3	Y	6,532,597	6,577,903	45,306	M	Duplication	-
505-3	Y	21,231,039	21,333,110	102,071	M	Duplication	-
554-3	X	118,460,470	118,487,729	27,259	M	Deletion	-
670-3	Y	22,243,114	22,489,344	246,230	M	Duplication	-
681-3	14	92,422,164	92,443,907	21,743	M	Duplication	TRIP11
681-3	2	112,530,696	112,579,331	48,635	M	Duplication	ANAPC1
681-3	20	756,842	791,208	34,366	M	Deletion	-
681-3	3	120,614,922	120,645,266	30,344	M	Deletion	STXBP5L
625-3	17	20,801,835	20,893,760	91,925	M	Duplication	-
659-3	2	21,438,794	21,462,096	23,302	M	Duplication	-
659-3	7	79,927,625	79,972,710	45,085	M	Deletion	-
659-3	7	80,299,710	80,354,513	54,803	M	Deletion	CD36
678-3	16	68,284,483	68,376,819	92,336	F	Duplication	SLC7A6,PLA2G15,SLC7A6OS,PRMT7
517-3	16	843,861	1,162,728	318,867	F	Duplication	C1QTNF8,LMF1,GNG13,SOX8,SSTR5,CHTF18,PRR25
517-3	16	2,088,391	2,415,016	326,625	F	Duplication	BRICD5,RNPS1,RAB26,TRAF7,SLC9A3R2,PKD1,E4F1,ECI1, ABCA3,PGP,CASKIN1,NTHL1,MLST8,TSC2,DNASE1L2
517-3	18	29,638,649	29,677,400	38,751	F	Duplication	RNF138,RNF125

517-3	7	4,015,113	4,685,115	670,002	F	Duplication	SDK1
517-3	X	81,298,618	81,338,460	39,842	F	Deletion	-
672-3	11	129,769,926	129,815,519	45,593	M	Duplication	PRDM10
672-3	21	22,941,489	23,009,531	68,042	M	Duplication	-
672-3	4	71,227,646	71,250,769	23,123	M	Deletion	SMR3B,SMR3A
672-3	Y	25,415,633	25,618,373	202,740	M	Deletion	DAZ3,DAZ2,DAZ4
508-3	5	24,949,269	24,990,295	41,026	M	Duplication	-
565-3	5	96,910,556	96,935,306	24,750	M	Deletion	-
565-3	6	169,508,876	169,539,121	30,245	M	Deletion	-
565-3	X	90,817,396	90,889,532	72,136	M	Deletion	-
565-3	Y	16,160,929	16,189,527	28,598	M	Duplication	VCY,VCY1B
530-3	20	31,988,157	32,009,801	21,644	M	Duplication	SNTA1,CDK5RAP1
530-3	3	15,687,586	15,710,397	22,811	M	Deletion	ANKRD28
515-3	12	47,262,280	47,330,558	68,278	M	Duplication	-
515-3	15	41,585,043	41,617,637	32,594	M	Duplication	OIP5
537-3	19	23,895,015	23,971,507	76,492	M	Deletion	ZNF681
537-3	7	27,497,389	27,537,776	40,387	M	Deletion	-
685-3	15	42,418,261	42,447,236	28,975	M	Deletion	PLA2G4F
685-3	19	37,772,900	37,803,996	31,096	M	Deletion	-
685-3	22	47,901,616	47,922,809	21,193	M	Duplication	-
685-3	6	68,342,496	68,410,749	68,253	M	Duplication	-
685-3	X	8,289,192	8,338,653	49,461	M	Deletion	-
646-3	5	120,872,438	120,895,959	23,521	M	Deletion	-
646-3	6	169,508,876	169,539,121	30,245	M	Deletion	-
560-3	6	80,110,296	80,354,297	244,001	M	Duplication	LCA5,SH3BGRL2
560-3	6	80,884,791	80,997,902	113,111	M	Duplication	BCKDHB
532-3	10	133,592,188	133,652,778	60,590	M	Duplication	-
532-3	17	17,444,603	17,476,218	31,615	M	Duplication	PEMT
532-3	3	159,562,374	159,641,399	79,025	M	Duplication	SCHIP1,IQCJ-SCHIP1
532-3	6	169,508,876	169,539,121	30,245	M	Deletion	-
608-3	14	93,363,869	93,408,980	45,111	M	Duplication	ITPK1,CHGA
608-3	3	2,352,000	2,434,492	82,492	M	Deletion	CNTN4
578-3	1	155,927,397	156,002,779	75,382	F	Duplication	SSR2,ARHGEF2
578-3	2	68,125,250	68,176,751	51,501	F	Deletion	-
578-3	7	7,953,108	7,999,416	46,308	F	Deletion	-
578-3	7	146,329,136	146,362,505	33,369	F	Deletion	CNTNAP2
578-3	8	5,722,189	5,784,231	62,042	F	Deletion	-
548-3	15	91,145,609	91,172,887	27,278	M	Deletion	CRTC3
550-3	14	106,291,501	106,319,497	27,996	M	Duplication	-
550-3	2	209,682,592	209,703,720	21,128	M	Deletion	-
550-3	7	152,508,063	153,499,963	991,900	M	Duplication	ACTR3B

550-3	X	143,651,892	143,748,641	96,749	M	Duplication	-
550-3	Y	22,311,731	22,489,344	177,613	M	Duplication	-
540-3	16	77,191,477	77,227,551	36,074	M	Duplication	MON1B
540-3	6	9,965,790	10,001,901	36,111	M	Deletion	-
540-3	Y	27,653,382	27,809,359	155,977	M	Deletion	CDY1B,CDY1
664-3	X	47,850,294	48,230,012	379,718	M	Duplication	SPACA5,SSX5,SSX3,SSX1,ZNF630,ZNF182,SPACA5B
664-3	Y	26,513,714	26,587,395	73,681	M	Deletion	-
682-3	6	162,296,842	162,359,917	63,075	M	Duplication	PARK2
682-3	7	43,361	705,271	661,910	M	Duplication	PDGFA,FAM20C,PRKAR1B,LOC100288524
549-3	16	16,295,901	16,855,348	559,447	M	Deletion	ABCC6,NOMO3
677-3	12	99,996,714	100,080,217	83,503	M	Deletion	ANKS1B,FAM71C
677-3	13	43,587,160	43,608,103	20,943	M	Duplication	DNAJC15
677-3	X	115,862,466	116,006,048	143,582	M	Deletion	-
511-3	2	186,884,124	187,008,504	124,380	M	Deletion	-
511-3	5	123,700,908	123,883,512	182,604	M	Duplication	-
511-3	9	28,464,218	28,596,286	132,068	M	Deletion	LINGO2
511-3	X	147,489,229	147,529,551	40,322	M	Duplication	-
686-3	10	18,240,592	18,313,842	73,250	M	Deletion	SLC39A12
686-3	4	27,394,986	27,415,922	20,936	M	Deletion	-
686-3	9	119,547,311	119,567,789	20,478	M	Deletion	ASTN2
521-3	16	14,357,944	14,386,767	28,823	M	Duplication	MKL2
647-3	1	79,572,639	79,967,028	394,389	M	Deletion	-
647-3	17	80,845,676	80,969,149	123,473	M	Duplication	TBCD,B3GNTL1
667-3	14	83,564,570	83,593,839	29,269	M	Duplication	-
667-3	3	192,367,797	192,418,953	51,156	M	Deletion	FGF12
667-3	4	77,393,565	77,442,934	49,369	M	Duplication	SHROOM3
567-3	11	25,664,801	25,738,322	73,521	M	Deletion	-
567-3	2	53,148,333	53,290,713	142,380	M	Deletion	-
567-3	4	122,480,241	122,577,042	96,801	M	Duplication	-
567-3	X	31,805,650	31,959,887	154,237	M	Deletion	DMD
493-3	10	78,292,989	78,320,204	27,215	F	Deletion	C10orf11
493-3	X	153,822,886	153,843,460	20,574	F	Deletion	-
683-3	2	233,651,280	233,673,273	21,993	F	Deletion	GIGYF2
683-3	7	90,464,909	90,723,377	258,468	F	Deletion	CDK14
683-3	8	5,543,465	5,608,834	65,369	F	Deletion	-
534-3	15	32,206,861	32,231,263	24,402	M	Duplication	-
534-3	4	71,227,646	71,250,769	23,123	M	Deletion	SMR3B,SMR3A
502-3	2	241,104,594	241,402,775	298,181	M	Duplication	GPC1
502-3	X	72,872,788	72,898,554	25,766	M	Duplication	CHIC1
631-3	17	19,521,054	19,563,766	42,712	F	Deletion	ALDH3A2
631-3	2	179,752,984	179,780,855	27,871	F	Deletion	CCDC141

631-3	7	9,437,710	9,465,942	28,232	F	Duplication	-
663-3	2	68,130,508	68,179,098	48,590	M	Deletion	-
663-3	6	169,494,891	169,539,121	44,230	M	Deletion	-
663-3	8	94,866,807	94,905,198	38,391	M	Deletion	-
663-3	X	72,332,078	72,353,391	21,313	M	Deletion	-
577-3	4	71,227,646	71,250,769	23,123	M	Deletion	SMR3B,SMR3A
577-3	9	468,705	489,338	20,633	M	Deletion	KANK1
577-3	Y	9,237,095	9,304,988	67,893	M	Deletion	TSPY4,TSPY3,TSPY1
577-3	Y	9,308,643	9,342,828	34,185	M	Deletion	TSPY4
668-3	11	124,133,810	124,231,918	98,108	M	Deletion	OR8G5,OR8G1,OR8D1,OR8D2
668-3	18	7,079,996	7,580,481	500,485	M	Duplication	LRRC30,LAMA1,PTPRM
668-3	2	134,077,947	134,151,644	73,697	M	Deletion	NCKAP5
668-3	22	46,946,802	47,354,509	407,707	M	Duplication	TBC1D22A,CERK,GRAMD4
539-3	2	194,568,253	194,601,354	33,101	M	Deletion	-
539-3	3	83,191,785	83,232,605	40,820	M	Duplication	-
607-3	7	85,624,564	85,673,895	49,331	M	Deletion	-
688-3	20	50,582,813	50,606,329	23,516	M	Deletion	-
574-3	19	58,791,213	58,813,839	22,626	M	Deletion	ZNF8
574-3	4	54,070,949	54,091,178	20,229	M	Deletion	SCFD2
574-3	8	5,057,783	5,323,185	265,402	M	Deletion	-
545-3	6	78,214,540	78,293,398	78,858	M	Deletion	-
545-3	7	125,147,776	125,168,006	20,230	M	Duplication	-
503-3	16	28,819,029	29,051,191	232,162	M	Deletion	ATXN2L,ATP2A1,NFATC2IP,SPNS1,RABEP2,SH2B1,LAT,TUFM,CD19
676-3	1	246,261,903	246,324,760	62,857	M	Deletion	SMYD3
676-3	12	95,622,380	95,665,403	43,023	M	Deletion	VEZT
676-3	15	42,421,124	42,447,236	26,112	M	Deletion	PLA2G4F
542-3	1	65,924,510	66,031,445	106,935	M	Duplication	LEPR
671-3	1	192,370,110	192,394,774	24,664	M	Deletion	-
671-3	Y	25,415,633	25,618,373	202,740	M	Deletion	DAZ3,DAZ2,DAZ4
691-3	1	208,513,516	208,556,206	42,690	M	Duplication	-
691-3	12	86,005,217	86,139,905	134,688	M	Duplication	-
691-3	9	115,714,003	115,746,828	32,825	M	Deletion	-
528-3	16	53,747,533	53,771,635	24,102	M	Deletion	FTO
528-3	3	22,039,451	22,103,146	63,695	M	Deletion	-
528-3	4	34,116,667	34,144,022	27,355	M	Deletion	-
528-3	6	127,607,555	127,654,281	46,726	M	Deletion	ECHDC1,RNF146
692-3	1	15,276,526	15,453,728	177,202	M	Duplication	KAZN
692-3	17	2,455,643	3,449,869	994,226	M	Duplication	CLUH,OR3A3,SPATA22,PAFAH1B1,OR1E1,OR1D5,OR1E2,OR3A1,OR3A2,OR1D2,OR1A2,OR1A1,RAP1GAP2,OR1G1,TRPV3,ASPA
692-3	2	212,057,149	212,142,292	85,143	M	Duplication	-
692-3	6	169,508,876	169,539,121	30,245	M	Deletion	-

485-3	10	87,338,403	87,359,978	21,575	M	Deletion	GRID1
485-3	11	11,179,056	11,296,482	117,426	M	Deletion	GALNT18
626-3	21	22,485,241	22,526,967	41,726	M	Deletion	NCAM2
636-3	14	40,727,363	40,780,133	52,770	M	Duplication	-
538-3	1	101,104,505	101,132,410	27,905	M	Deletion	-
538-3	X	109,315,806	109,340,564	24,758	M	Deletion	TMEM164
562-3	10	135,377,075	135,400,250	23,175	F	Deletion	SYCE1
562-3	2	23,694,732	23,726,237	31,505	F	Deletion	KLHL29
562-3	3	128,340,747	128,367,730	26,983	F	Duplication	RPN1
562-3	3	136,633,504	136,664,731	31,227	F	Duplication	NCK1
562-3	6	100,423,832	100,467,829	43,997	F	Duplication	MCHR2
562-3	9	33,876,490	33,951,019	74,529	F	Duplication	UBAP2,UBE2R2
622-3	4	127,751,482	127,777,218	25,736	M	Deletion	-
623-3	19	55,435,082	55,754,138	319,056	M	Duplication	TNNT1,NLRP7,PPP6R1,NLRP2,PPP1R12C,SYT5,TNNI3, RDH13,EPS8L1,GP6,DNAAF3,TMEM86B,PTPRH
623-3	Y	27,410,703	27,607,527	196,824	M	Duplication	-
576-3	10	96,581,094	96,626,365	45,271	M	Deletion	CYP2C19
576-3	21	43,837,058	43,893,628	56,570	M	Duplication	UBASH3A,RSPH1
576-3	9	9,473,063	9,572,759	99,696	M	Deletion	PTPRD
576-3	9	109,270,732	109,310,538	39,806	M	Duplication	-
662-3	11	85,774,746	85,845,045	70,299	M	Duplication	PICALM
662-3	4	81,335,556	81,367,124	31,568	M	Duplication	C4orf22
552-3	11	80,696,716	80,801,906	105,190	M	Duplication	-
552-3	5	56,498,189	56,574,179	75,990	M	Duplication	GPBP1
552-3	5	178,679,462	178,790,851	111,389	M	Deletion	ADAMTS2
552-3	7	14,122,604	14,199,001	76,397	M	Deletion	DGKB
552-3	X	72,319,907	72,353,391	33,484	M	Deletion	NAP1L6
658-3	1	5,734,013	5,767,396	33,383	M	Deletion	-
658-3	5	151,292,056	151,349,089	57,033	M	Duplication	GLRA1
544-3	10	112,577,721	112,632,630	54,909	M	Duplication	PDCD4,RBM20
544-3	2	214,391,134	214,424,922	33,788	M	Duplication	SPAG16
520-3	7	19,980,047	20,098,212	118,165	M	Deletion	-
520-3	7	111,218,682	111,319,190	100,508	M	Duplication	-
520-3	8	53,492,860	53,549,179	56,319	M	Duplication	RB1CC1
519-3	2	241,544,852	241,572,469	27,617	M	Deletion	GPR35
519-3	X	153,408,930	153,438,781	29,851	M	Duplication	OPN1LW
535-3	2	35,758,580	35,795,100	36,520	M	Deletion	-
535-3	3	164,088,939	164,113,222	24,283	M	Deletion	-
514-3	11	11,116,025	11,187,898	71,873	M	Duplication	-
514-3	16	17,940,666	17,963,706	23,040	M	Duplication	-
514-3	5	96,910,556	96,935,306	24,750	M	Deletion	-
514-3	5	153,412,185	153,531,977	119,792	M	Duplication	FAM114A2,MFAP3

523-3	2	75,820,691	75,919,242	98,551	M	Duplication	MRPL19,GCFC2
523-3	3	285,783	318,812	33,029	M	Deletion	CHL1
523-3	8	89,774,917	89,993,041	218,124	M	Deletion	-
611-3	13	89,365,687	89,574,513	208,826	M	Duplication	-
611-3	7	37,913,050	38,246,663	333,613	M	Duplication	NME8,STARD3NL,SFRP4,EPDR1
611-3	X	32,548,066	32,603,018	54,952	M	Deletion	DMD
611-3	X	125,884,392	125,957,401	73,009	M	Deletion	CXorf64
609-3	11	10,515,751	10,537,581	21,830	M	Duplication	AMPD3,RNF141,MTRNR2L8
609-3	8	54,058,999	54,124,081	65,082	M	Duplication	-
609-3	Y	26,504,496	26,592,401	87,905	M	Deletion	-
687-3	5	96,910,556	96,935,306	24,750	M	Deletion	-
489-3	14	94,555,466	94,587,345	31,879	M	Duplication	IFI27,IFI27L1
489-3	17	18,692,538	18,726,389	33,851	M	Deletion	TVP23B
489-3	4	64,569,828	65,118,025	548,197	M	Duplication	-
610-3	X	82,973,565	82,997,765	24,200	M	Duplication	-
533-3	22	50,000,220	50,049,643	49,423	M	Duplication	-
693-3	2	74,892,914	74,990,885	97,971	M	Duplication	SEMA4F
693-3	5	164,041,749	164,131,456	89,707	M	Duplication	-
693-3	X	134,384,557	134,802,358	417,801	M	Duplication	DDX26B,ZNF75D,ZNF449
693-3	Y	9,492,812	9,522,716	29,904	M	Deletion	-
694-3	17	18,562,720	18,590,815	28,095	M	Deletion	ZNF286B
694-3	4	34,658,484	34,685,352	26,868	M	Deletion	-
694-3	4	48,825,457	49,093,773	268,316	M	Duplication	OCIAD2,CWH43,OCIAD1
694-3	4	94,144,621	94,172,410	27,789	M	Deletion	GRID2
694-3	5	19,409,085	19,430,309	21,224	M	Deletion	-
694-3	6	169,508,876	169,539,121	30,245	M	Deletion	-
694-3	9	28,491,679	28,630,598	138,919	M	Deletion	LINGO2
656-3	1	25,583,489	25,756,453	172,964	M	Duplication	RHCE,RHD,TMEM50A
656-3	14	87,355,480	87,446,496	91,016	M	Deletion	-
656-3	7	4,490,966	4,525,949	34,983	M	Deletion	-
628-3	20	8,913,944	8,976,141	62,197	M	Duplication	-
628-3	5	14,206,863	14,253,937	47,074	M	Deletion	TRIO
628-3	9	115,714,003	115,746,828	32,825	M	Deletion	-
689-3	14	62,849,708	62,960,957	111,249	M	Duplication	-
638-3	14	41,546,521	41,744,165	197,644	F	Deletion	-
638-3	15	42,421,124	42,447,236	26,112	F	Deletion	PLA2G4F
546-3	21	23,526,014	23,587,148	61,134	F	Deletion	-
546-3	5	151,263,430	152,000,379	736,949	F	Duplication	NMUR2,GLRA1
546-3	6	169,508,876	169,539,121	30,245	F	Deletion	-
546-3	9	6,518,780	6,655,056	136,276	F	Deletion	GLDC

Supplementary Table 2: Summary statistics of stringent CNVs larger than 20 kb

	**ASD Probands**	**Parents**	**OPGP Controls**
Samples	100	200	873
Males/Females	90/10	100/100	477/396
#Stringent CNVs	1,638	3,260	15,872
Mean CNVs/sample ± SD[a]	16.38±4.00	16.30±3.84	18.18±4.41
Median	16	16	18
Mean CNV size (kb) ± SD[b]	99.04±213.32	89.41±181.08	90.27±166.64
Median CNV size (kb)	44.54	43.98	42.11
%Gain/%Loss	43.1%/56.9%	44.4%/55.6%	43.2%/56.8%
#CNVs > 1Mb (%)	14 (0.85%)	21 (0.64%)	116 (0.73%)
#CNVs 100kb-1Mb (%)	353 (21.55%)	658 (20.18%)	3190 (20.10%)
#CNVs 20 kb-100kb (%)	1,271 (77.60%)	2,581 (79.18%)	12,566 (79.17%)

[a] There is no significant difference between the mean number of CNV calls in ASD probands and their parents (p=0.8669 using an unpaired two-tailed Fisher's exact test). We do notice a significant difference between the number of calls noted in ASD cases and OPGP controls run on the same array (p=0.0001). We suspect that this is due to some batch effect and is not representative of some biological difference between cases and controls as the number of calls in controls is higher.

[b] There is no difference between the mean CNV size in ASD probands and either their parents or OPGP controls (p=0.6831 and p=0.6292, respectively).

Supplementary References

1. Pinto D, Darvishi K, Shi X, Rajan D, Rigler D, Fitzgerald T, Lionel AC, Thiruvahindrapuram B, Macdonald JR, Mills R, Prasad A, Noonan K, Gribble S, Prigmore E, Donahoe PK, Smith RS, Park JH, Hurles ME, Carter NP, Lee C, Scherer SW, Feuk L: **Comprehensive assessment of array-based platforms and calling algorithms for detection of copy number variants.** *Nature biotechnology* 2011, **29:**512-520.
2. Darvishi K: **Application of Nexus copy number software for CNV detection and analysis.** *Current protocols in human genetics / editorial board, Jonathan L Haines [et al]* 2010, **Chapter 4:**Unit 4 14 11-28.
3. Downey T: **Analysis of a multifactor microarray study using Partek genomics solution.** *Methods in enzymology* 2006, **411:**256-270.
4. Purcell S, Neale B, Todd-Brown K, Thomas L, Ferreira MA, Bender D, Maller J, Sklar P, de Bakker PI, Daly MJ, Sham PC: **PLINK: a tool set for whole-genome association and population-based linkage analyses.** *American journal of human genetics* 2007, **81:**559-575.
5. Costain G, Lionel AC, Merico D, Forsythe P, Russell K, Lowther C, Yuen T, Husted J, Stavropoulos DJ, Speevak M, Chow EW, Marshall CR, Scherer SW, Bassett AS: **Pathogenic rare copy number variants in community-based schizophrenia suggest a potential role for clinical microarrays.** *Human molecular genetics* 2013, **22:**4485-4501.
6. Altshuler DM, Gibbs RA, Peltonen L, Dermitzakis E, Schaffner SF, Yu F, Bonnen PE, de Bakker PI, Deloukas P, Gabriel SB, Gwilliam R, Hunt S, Inouye M, Jia X, Palotie A, Parkin M, Whittaker P, Chang K, Hawes A, Lewis LR, Ren Y, Wheeler D, Muzny DM, Barnes C, Darvishi K, Hurles M, Korn JM, Kristiansson K, Lee C, McCarrol SA, Nemesh J, Keinan A, Montgomery SB, Pollack S, Price AL, Soranzo N, Gonzaga-Jauregui C, Anttila V, Brodeur W, Daly MJ, Leslie S, McVean G, Moutsianas L, Nguyen H, Zhang Q, Ghori MJ, McGinnis R, McLaren W, Takeuchi F, Grossman SR, Shlyakhter I, Hostetter EB, Sabeti PC, Adebamowo CA, Foster MW, Gordon DR, Licinio J, Manca MC, Marshall PA, Matsuda I, Ngare D, Wang VO, Reddy D, Rotimi CN, Royal CD, Sharp RR, Zeng C, Brooks LD, McEwen JE: **Integrating common and rare genetic variation in diverse human populations.** *Nature* 2010, **467:**52-58.
7. Lou H, Li S, Yang Y, Kang L, Zhang X, Jin W, Wu B, Jin L, Xu S: **A map of copy number variations in Chinese populations.** *PloS one* 2011, **6:**e27341.
8. Xu H, Poh WT, Sim X, Ong RT, Suo C, Tay WT, Khor CC, Seielstad M, Liu J, Aung T, Tai ES, Wong TY, Chia KS, Teo YY: **SgD-CNV, a database for common and rare copy number variants in three Asian populations.** *Human mutation* 2011, **32:**1341-1349.
9. Uddin M, Tammimies K, Pellecchia G, Alipanahi B, Hu P, Wang Z, Pinto D, Lau L, Nalpathamkalam T, Marshall CR, Blencowe BJ, Frey BJ, Merico D, Yuen RK, Scherer SW: **Brain-expressed exons under purifying selection are enriched for de novo mutations in autism spectrum disorder.** *Nature genetics* 2014.
